# ‘If I am playing football, I forget that I have this virus’: the challenges and coping strategies of adolescents with perinatally acquired HIV in KwaZulu-Natal, South Africa

**DOI:** 10.1186/s12879-022-07780-x

**Published:** 2022-10-21

**Authors:** Marian Loveday, Jennifer Furin, Sindisiwe Hlangu, Thabile Mthethwa, Tasneem Naidoo

**Affiliations:** 1 HIV and other Infectious Diseases Research Unit, South African Medical Research Council, 491 Peter Mokaba Ridge Road, Overport, Durban, KwaZulu-Natal South Africa; 2 Centre for Health Systems Research & Development, University of the Free State, Bloemfontein, South Africa; 3 CAPRISA-MRC HIV-TB Pathogenesis and Treatment Research Unit, Doris Duke Medical Research Institute, University of KwaZulu-Natal, Durban, South Africa; 4 Department of Global Health and Social Medicine, Harvard Medical School, Boston, MA 02115 USA; 5 Department of Health, RK Khan Hospital HAST Unit, Durban, KwaZulu-Natal South Africa

**Keywords:** South Africa, HIV, Adolescents, Antiretroviral therapy, Adherence

## Abstract

**Background:**

Adolescents who have acquired HIV perinatally (ALHIV) face unique challenges in taking lifelong antiretroviral therapy (ART), but little is known about what factors affect their adherence over the course of their lifelong treatment journey.

**Methods:**

We conducted a qualitative study with ALHIV who had periods of poor adherence to ART in KwaZulu-Natal, South Africa using Participant-generated Visual Methodologies (PVM). Participants used photography to represent their perspectives and experiences.

**Results:**

14 individuals participated in the research process. We developed a framework and identified four social domains which combined with the adolescent’s own experiences and sense of self to either support or undermine adherence. Periods of non-adherence were reported by all participants. Participants described the importance of supportive relationships and households as well as the benefits of ART as supporting adherence. The fear of inadvertent disclosure of their HIV status and the side-effects of ART were barriers to adherence. Possible interventions to support adolescents in their treatment journey are identified.

**Conclusions:**

Current models of adherence support fail to address the challenges to lifelong therapy ALHIV face. Ongoing education and honest communication with health care providers, interventions that build resilience together with peer support, have the potential to improve adherence in ALHIV.

**Supplementary Information:**

The online version contains supplementary material available at 10.1186/s12879-022-07780-x.

## Background

Adolescence, defined by the World Health Organization as the time period when a person is between 10 and 19 years old, is a transitional stage from child- to adulthood which has major health implications [1, 2]. Nearly two million adolescents in sub-Saharan Africa (SSA) are living with HIV [3]. Adolescents living with HIV (ALHIV) include those who have acquired HIV perinatally and those who have acquired HIV through sexual intercourse. Of the global population of ALHIV acquired perinatally, up to 80% live in SSA, with many living in South Africa [4]. Sustaining optimal antiretroviral therapy (ART) adherence for ALHIV has emerged as a major challenge and there is substantial evidence that ALHIV acquired perinatally experience greater challenges to treatment adherence compared to both younger children and adults [5–7]. Nonadherence is associated with treatment failure, emergence of drug resistance, disease progression and high morbidity [8, 9].

This adolescent period is one of orientation and discovery as questions of independence, identity and a sense of self emerge. It is a time of heightened vulnerability as choices about future, friendship, sexuality, gender identity, and substance use are negotiated, all of which can have an impact on adolescent health, especially among ALHIV [10, 11]. With an evolving capacity to make health decisions and participate in promoting their own welfare [12], the needs and vulnerabilities of early, middle and late adolescents vary as do optimal models for chronic disease adherence support [13].

Comparatively little is known about what affects the HIV care continuum in adolescents compared with adult populations [14], especially among adolescents who acquired HIV perinatally [14]. There is a pressing need to better describe the treatment journeys of adolescents living with HIV, with an eye toward improving adherence and retention in care. We report the results of a qualitative study we undertook with ALHIV who were on ART about their illness and the enablers and barriers to ongoing engagement with adherence and the HIV services that they encountered on their treatment journeys.

## Methods

### Study design

This qualitative study used Participant-generated Visual Methodologies (PVM) which enables participants to use art, drawing or photography to represent their perspectives and experiences through self-captured images [15, 16]. PVM has been shown to be effective when working with populations that might not responds to traditional, questionnaire-driven interviews and has been used to capture the experiences of children and adolescents with HIV in South Africa [17, 18]. Using PVM, study participants were asked to capture images representing their experiences of living with HIV, together with the challenges encountered and support they received during their treatment journey that impacted their adherence and retention in care. This is described in more detail in the data collection section below.

In South Africa, due to conflicting statutes, regulations and ethical guidelines about who provides informed consent for adolescent involvement in health research, some ethics committees are reluctant to grant a waiver of parental consent for adolescents < 18 years. Fearing parental consent could exclude those adolescents most vulnerable and at-risk, we requested a waiver of parental consent, which was only granted for those ≥ 16 years old. Hence, we enrolled participants 16–19 years old. Enrolment was limited to those with sub-optimal retention, defined as not taking ART for > 30 days in the last 2 months, as documented in the clinical file.

### Study setting and population

The study was conducted at RK Khan regional hospital, in a peri-urban setting in KwaZulu-Natal South Africa over 3 months, from 1 December 2021–28 February 2022. RK Khan Hospital has a large ART clinic providing HIV management for the local population as well as serving as a referral centre for a broader area. Between January 1 and December 31, 2021, 15 ALHIV were started on ART at the hospital. Currently RK Khan ART clinic manages > 320 ALHIV.

### Data collection

Seventy-eight (78) ALHIV who met the study criteria were identified in the ART clinic register. Only 19 (25%) participants could be contacted, of whom three refused participation and one had relocated. Fifteen participants signed consent and were enrolled, but one did not arrive, leaving 14 to participate in the study. As part of the informed consent process participants were told about the study team members’ interest in the topic matter and that they were employed as full-time researchers.

Two training workshops with seven participants in each were facilitated by two authors (SH and TM), one of whom (SH) has a decade of qualitative research experience. At the training workshops, which lasted one and a half hours, the background and rationale of the study were described, and each participant was provided with a cell phone with which to take pictures, and painting and/drawing materials. The idea of capturing experiences in an appropriate manner using either a cell phone or drawing or painting was introduced. Participants were provided with a one-page ‘Participant Guideline’ document to minimise risk and to explain how the photographs should be taken (This is included in Additional file 1: Annexure 1).

The week after the training course, participants captured images that symbolized their experiences of living with HIV in their community, together with something(s) that symbolized the challenges encountered and support they received during their treatment journey that impacted their adherence. After a week, they attended a Focus Group Discussion (FGD) led by two female authors (SH and TM) at which they presented and discussed their pictures in more depth. The FGDs were conducted in the local language (isiZulu) and lasted between two and a half and three hours. The FGDs were audio recorded and then translated and transcribed. Field notes were not kept or analysed. (Details of the methodology are described in Additional file 1: Annexure 1). Data analysis was conducted by two authors (ML and JF).

### Data analysis and theoretical framework

**Fig. 1:**
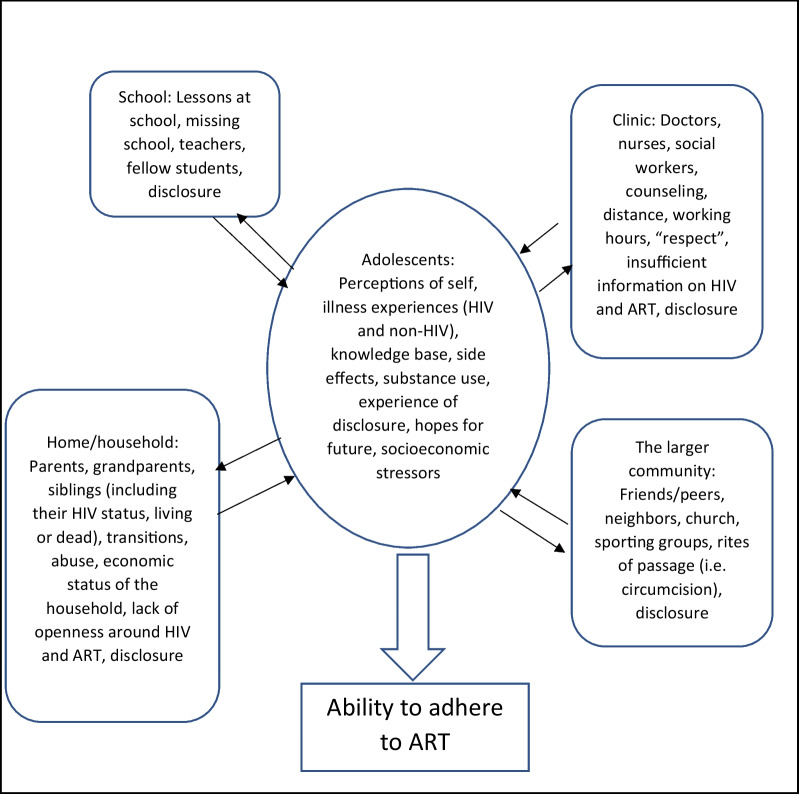
Theoretical framework for describing factors affecting adolescent identity and adherence to ART

Data (including the FGD recordings and the images shared by the participants) were analyzed for theme and content. The life-course perspective framework for adolescent health developed by Sawyer and colleagues was used and adapted for the data analysis in this paper [19]. In this framework, the health behaviors of adolescents are seen as being impacted by social determinants and institutions that may enable or impede health as the adolescent negotiates his/her changing identity [20]. We combined the life-course perspective framework with a dynamic theory of ART adherence developed by Eshun-Wilson and colleagues to further inform our data analysis [21]. Finally, we also used elements of theory from a social-ecological model [22] to supplement our data analysis, including the concept of a “social domain”. A social domain is defined as a communicative context that influences and is influenced by behaviors [23] and perceptions [24]. In our theoretical framework, the behaviour of interest was adherence to ART and the social domains act upon adherence-related behaviours after being processed by, reflected on, and incorporated into adolescent perceptions of themselves [25]. The final theoretical framework based on important social domains in the adolescent world which we used to guide our data analysis is included in Fig. 1.

### Reflexivity

As part of the important practice of reflexivity, which is essential in qualitative research [26], our team acknowledges that as some of us are health care providers, we may have had biases regarding the experiences of the individual participants which led to us overly focusing on biomedical aspects of adolescent experiences with ART. As an all-female research team, we also recognize that our gender may have had an impact on the interview process, data analysis, and write-up of the results which could have led to marginalization or misunderstanding of male perspectives and voices. Furthermore, none of us are adolescents and this may also have impacted the ability of participants to communicate with us in an open and trusting fashion. We discussed these areas with one another and how they might have biased our analyses and results throughout the research project.

## Results

### Participant characteristics

The age of the participants ranged from 16 to 19 years and nine of the 14 participants were males (Table 1). All 14 study participants had acquired HIV perinatally. Six of the fourteen study participants had mothers who were still alive and only three had fathers who were present in their lives. One participant had never met his father but had spoken to him on the phone.

**Table 1:** Demographic and household characteristics of participants

#	Gender	Age	Relationship status with parents and household composition	Household members’ HIV and ART status
1	M	16	Lives with his mother and abusive stepfather. His relationship with his mother is not easy due to the stepfather. He has never met his father, but they have spoken on the phone	Mother on ART. Stepfather unaware of mother and participants HIV and ART status
2	M	19	His mother is deceased, so he lives with grandmother and aunt. His father has remarried, but supports him and during the holidays the participant stays with him	Grandmother and aunt on ART Father is also on ART
3	F	18	Mother died at birth of the participant. She lives with siblings, cousins and grandfather. The participant has never had any contact with father. She doesn’t know if he is alive or not	Participant is the only one in the household on ART
4	F	16	She lives with her mother, father and siblings and has a good relationship with both her parents	Mother and father are on ART
5	M	19	His mother is deceased, so he lives with grandmother, siblings and cousins who also lost their mother to HIV. He has met his father, but has no regular contact with him. His father does not support him and has no part in his life	Father is on ART. Some of the cousins and siblings in the household are on ART
6	F	18	Her mother and father are both deceased. She lives with her grandmother but does have contact with her father’s side of the family	Only the participant on ART
7	M	17	When both mother and father died of HIV he was taken into an orphanage. He is now looked after by a foster family	Only the participant on ART
8	M	17	His mother is a domestic worker, so he lives with his grandmother. His mother has never spoken about his father, so he doesn’t know his father and whether he is alive or not	Mother is on ART
9	F	17	Both parents are alive, but have no contact with their daughter and do not support her in any way. Her mother lives with boyfriend and since birth has failed to look after her. Participant lives with her grandmother and aunt	Mother and aunt are both HIV-positive. Mother not taking treatment
10	M	17	He lives with his mother. His father is in prison and has no contact with his son	Mother is HIV-positive on ART
11	M	16	He lives with his mother. His father died of HIV	Mother is HIV-positive on ART
12	M	18	He lives with his mother. His father died of HIV	Mother is HIV-positive on ART
13	F	16	She was abandoned by her mother as an infant and has never met her. She has never met her father and knows nothing about him. She lives with a guardian	She does not know the HIV status of her guardian
14	M	19	His mother is deceased, but his father is alive. He lives with his father and stepmother	Father and stepmother on ART and supportive of him

ART: antiretroviral therapy

### Thematic overview

Adolescents who participated in this study recalled three periods of time in terms of their HIV status and ART. From the age of birth until 11 years, they reported that they relied on their caregivers to manage their ART. In the period between 12 and 14 years, they reported experiencing disclosure of their HIV status to them and found that their reaction to this disclosure coloured their ART choices. In the period between 15 and 19 years, they experimented with not taking ART to determine whether they really needed the medication or not and their adherence was affected by their experience of ART and its side effects.

One participant described his HIV treatment journey in the following way:

*‘I didn’t know that I had HIV. I wasn’t taking any treatment. When I was 5/6 years old my mother got to the hospital and died. At home, I was told that I must go to the hospital to do blood tests. When I got there, I was also diagnosed with HIV’……..’I used to feel tired a lot at school, but they were not telling me what the pills I was taking were for’……’It was my mother’s death on the one hand and the pills on the other, it was a lot and eventually I stopped taking the pills and I started getting sick. I went to hospital and it was decided that I had to be told what the pills were for, I was 16 at that time. I was then told what the pills are for. I accepted it and I took the pills.’*

Another participant described his treatment journey and reason for adherence as follows:

*‘My family didn’t tell me that I have HIV. I was taking it [ART] when I was very small, at 6 months old. My father told me when I was older I was HIV positive’…..’No. I was taking the pills but not every day, and so that resulted in me getting TB. So, I was admitted in hospital for almost 6 months at Albert Luthuli hospital.’*

*When asked if he ever thought of defaulting again, his response was:*

*‘No…because of what I saw in hospital, what I saw frightened me. People die in hospital.’*

The data revealed four social domains which impacted adolescent adherence to ART due to their influence on adolescent perceptions of self. These domains were: (1) the household; (2) the school; (3) the larger community; and (4) the clinic. Experiences and relationships in each of these areas together with unfolding knowledge were internalized and processed by each adolescent as they negotiated their changing identity in the world. These domains combined with the adolescent’s own experiences and sense of self led to adaptation and the emergence of coping mechanisms which either supported engagement with HIV services (including ART) or acted as barriers to engagement. Each of these domains will be explored in more detail below, including the enablers and barriers they presented for adolescent adherence to ART.

#### Social domain 1: the household

The household in which the adolescent resided exerted important influences on his/her ability to engage in HIV care and adhere to ART. In childhood, adherence to ART was totally dependent on the care and support of individuals within the household. In their teenage years participants became increasingly responsible for engaging with HIV care, but multiple individuals within the household, including parents, grandparents and siblings, remained significant in either supporting or challenging adherence.

##### The support of household/family members

**Fig. 2:**
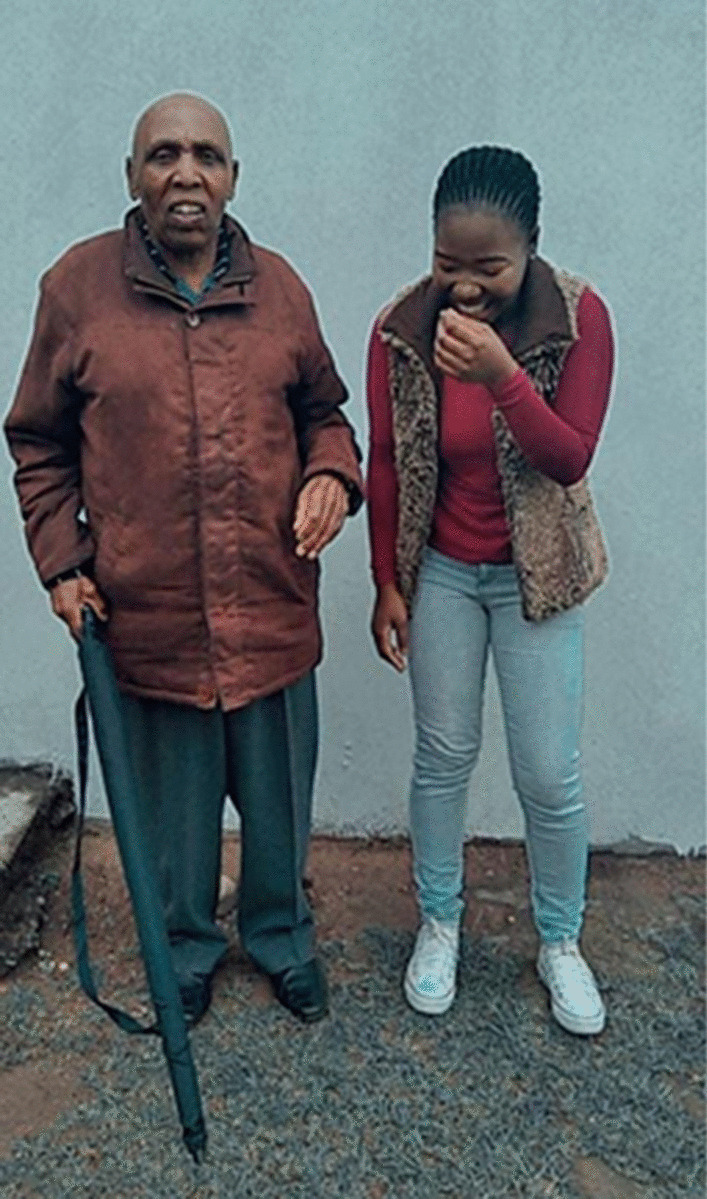
A picture of a supportive relative as a facilitator of adherence

When household members were positive about the adolescents’ engagement in care, this was reported to be a significant contributor to adherence to ART. As an example, one participant lost her mother at birth. She and her siblings live with their grandfather and their cousins, who also lost their mother to HIV. She shared a photo with the group (Fig. 2) of her grandfather and described how he often reminded her to take her ART.

A number of participants whose mothers were still alive spoke of how their mother’s support enabled adherence. Not only did their mothers remind them to take their ART daily, but they also picked up their adolescents’ medication at the clinic, so that the adolescent did not have to miss school. One participant, whose mother was living with HIV acknowledged her support. However, he reported that his siblings who were HIV-negative were not supportive of either of them. He chose to identify with his mother, and this helped him to remain engaged with HIV care, as reported below:

*‘That is what confused me, and I asked myself, “Why me?”, but as time went by, I understood that perhaps I have this ability to empathize with my mother’s pain and there is no one I care for more than I care for my mother.’*

##### Absent parents

A number of participants described the difficulties they experienced at home as barriers to adherence. This was particularly true of orphans or those with one or other absent parent who spoke of intermittent engagement with HIV services in childhood and unsupportive home circumstances during adolescence with little or no support, effective guidance, or supervision which impacted both their psychological and physical well-being. As can be seen in Table 1, five of the seven participants had lost their mothers and only three knew their father. One of these three, although he has spoken to his father on the phone, had never met him. One participant had great difficulty at home with an abusive stepfather who was unaware that both his wife and stepson were HIV-positive and on treatment. He became depressed, wanted to sleep the whole time, did not attend school or take his ART. This improved when he went to live with this grandmother. He recounted how his stepfather had chased him away from the house saying:

*‘….he always says to me that this house is not my home…..and then end up chasing me away….’*

Most participants expressed anger and bewilderment as to why their mothers had not prevented them from getting HIV through vertical transmission. One participant, whose mother died from HIV when she was 5 years old said:

*‘Sometimes I feel like my mother didn’t think of me. I don’t know why. I am the only one amongst my siblings who has it. I very much blame her. Even if I had a chance to ask her what happened, or even if she just had an opportunity to apologize to me…. because I believe that I need to speak with her about this, a lot.’*

Linked to this was the disclosure of participants’ HIV status to them and when and how this was communicated. Most participants were started on ART as children, but their caregivers, fearful of the child’s inadvertent disclosure of their HIV status and the stigma the child and household would face, only disclosed to the participants in their teenage years. Some participants who had been on ART as children stopped taking their medication regularly as they got older. One reported that when told by his mother that he had HIV, he was angry and stopped taking his treatment for a year.

##### Moving between different households

**Fig. 3:**
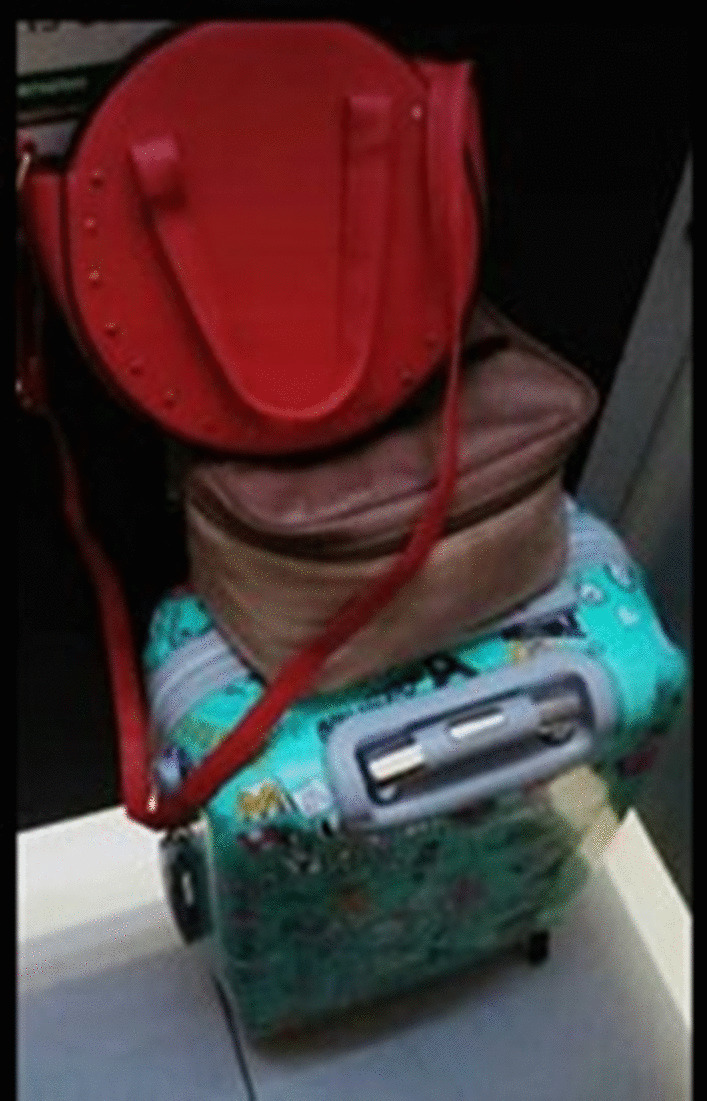
A photo of suitcases to illustrate travelling as a barrier to adherence

Not being at home, in their normal routine, eating their normal food with family who know they were on ART was also described as a barrier to care by some participants. One participant shared a photo of suitcases to illustrate travelling as a barrier to care (Fig. 3). He described how when he stays with the paternal side of his family there is a lot of sweet food. This sweet food makes him nauseous which makes taking ART more difficult. In addition, travelling was at times the reason he missed his hospital appointments. This participant described how, as a child, when staying at this house, there was no engagement with HIV services or adherence to ART.

##### Lack of household openness in discussing HIV and ART

Many participants described the lack of openness about HIV and ART at a household level and how this was a barrier to retention in care as no-one in the household knew their HIV status or supported them on their treatment journey. Two participant’s whose siblings were not HIV-infected expressed bewilderment as to why they were infected with HIV and the other siblings not. However, as they had never talked or asked their mother about this, their knowledge and understanding of HIV transmission remained limited and did not inform the importance of adherence.

##### The economic status of the household

Fearful of disclosure most participants did not go to their local clinic and needed money to get to the hospital ART clinic for their monthly medication. For those from unsupportive or poor households, a lack of money impacted on their accessing the clinic and their medication and was a barrier to adherence.

#### Social domain 2: the school

Adolescents who participated in the study reported spending most of their time in school, and thus the social domain of school had an impact on their ability to remain engaged in HIV care. Interactions with peers, interactions with teachers, the content of lessons, and missing school to attend clinic appointments were all important subthemes that emerged as potential enablers or barriers to adherence.

##### School content

A number of study participants described how difficult it is for them to negotiate discussions about HIV within the classroom and with fellow students, due to the judgemental attitudes of their class mates towards those who were HIV positive. One participant said that when HIV comes up in the classroom she just wants to go home. In relation to discussing HIV with his colleagues, one participant said:

*‘It’s disheartening because you have the knowledge that whatever they are saying is not entirely true. But you don’t say as they will ask where you are getting so much information from. They will just conclude that you are taking HIV treatment.’*

##### The fear of inadvertent disclosure, stigma and discrimination

For all participants, the fear of unintended disclosure of their HIV status at school and to people in the larger community was a major concern. This fear impacted their willingness to attend the clinic to collect their ART.

All participants described their anxiety about bumping into someone from school or someone they knew at the clinic. Three participants described bumping into a fellow pupil. One participant had a story prepared for when she encountered classmates at clinic and said she was visiting a sick child. Similarly, one described how he had a story prepared, and although he doesn’t have asthma, if anyone asks him why he is taking pills, he responds it is for his asthma. Another participant reported always going to the clinic with her grandmother so she was able to say she is accompanying her grandmother.

Fearful of stigma, rejection and gossip, two participants described the difficulties they experienced in ensuring no-one at school observed them taking their medication. One participant always packed his ART into a different container so that it couldn’t be recognised as ART and another participant reported:

*‘No, they would not see me. I would take my water bottle to the tap and just turn my back against them and swallow the pills, they would not see me.’*

Another participant described how since developing epilepsy during the Covid-pandemic, it was easier for him to take his ART:

*‘It’s a lot easier now because I also take pills for epilepsy, so, when I take pills, everyone just thinks I am taking pills for epilepsy.’*

##### ART clinic and missing class

One participant described that on the day he has to go the clinic to collect his medication he does not go to school as he cannot bear to explain why he is late. The next day he tells the teacher he was ill. In contrast, two other participants described how they get a letter from the clinic that they give to the teacher when they arrive late, so they never miss a days school due to a clinic visit.

One participant presented a photo of her and her class during the FGD, and described school as a barrier to care:

*‘In my class we were a group of girls, so, I never wanted to miss out on anything in class. So, making it to my clinic appointments was very difficult because I would always postpone to the following Monday or Tuesday and I just end up missing my appointments like that.’*

##### Relationships at school

One participant reported he was able to talk to his teacher, who was supportive of him, both as a person and as a person living with HIV. Another participant described a friend at school:

*‘There is a friend of mine who also has this virus, at break we sit together and just chat about this virus. We go as far as asking each other how the other is coping and we share ideas on how to tackle it. We also talk about ways to protect ourselves from people finding out that we have this virus.’*

However, as is reported in the section on relationships in the larger community below, many participants were unable to disclose their HIV status to their peers or discuss the challenges they encountered.

#### Social domain 3: the larger community

Other important institutions that promoted or detracted from adolescent engagement in HIV care were part of the larger communities in which they operated, including church, sports groups, peers/friends, and community events/rites of passage.

##### Relationships

**Fig. 4:**
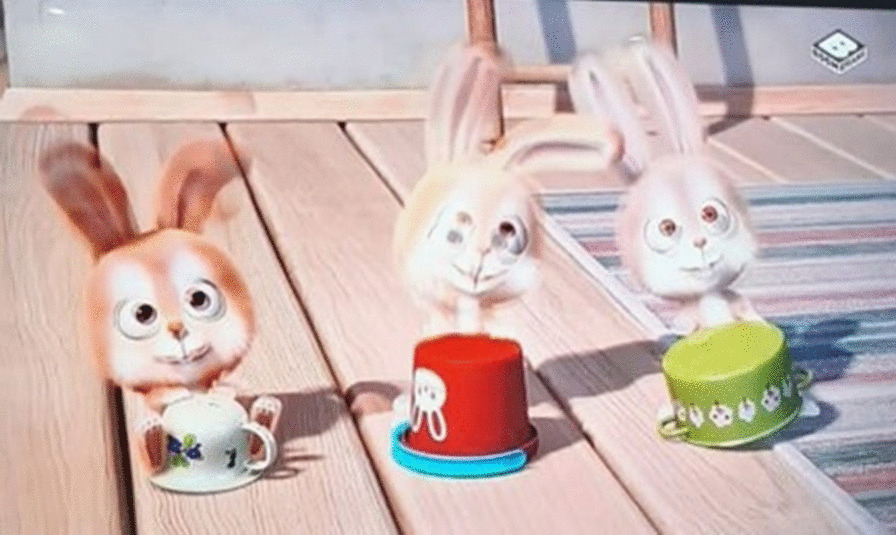
A photo of cartoon characters

All participants reported that their individual relationships and embarking on more intimate relationships with significant others were affected by their fear of inadvertent disclosure of their HIV status. They acknowledged the pain associated with this. Fearful of rejection and gossip, a couple of participants who were involved in intimate relationships, had not disclosed their HIV status to their partners. One participant took a photo of cartoon characters (Fig. 4) and described his rationale for taking the photo as follows:

*‘That’s my picture, it means that I like being indoors and watching cartoons. I don’t go out much.’*

One participant took a photo of his friends. The day he took the photo, he was walking around the neighbourhood when they came across a mobile HIV testing van. They all decided to get tested, but he refused:

*‘Yah, it’s painful to live with this virus. ……..I just ducked away, and I told them that I am not getting tested and that was my final word.’*

He went on to say that he has not told his friends about his status:

*‘Eyy, people talk too much, you can’t trust people. I don’t want them to know.’….. ‘I have friends who talk a lot, they talk too much. Sometimes when the topic about dating people with HIV arises, I always pretend as if I don’t have this virus.’…. ‘I don’t want people finding out that I have this virus.’*

Responding to the question as to why participants did not want to share their HIV status with their friends, another participant said:

*‘It’s because it’s not easy to tell people that you have HIV, because as he said, when you have a fall out with someone, they may go around telling people that you have HIV. People will then start distancing themselves from you because of the belief that you will transmit the virus to them.’*

All the participants agreed with this sentiment. One respondent added that his friends would only find out about his HIV status when he was dead and one participant stated that he would trust nobody with his HIV status except *‘his family, his bloodline*.’

##### Rites of passage

In South Africa, medical male circumcision has been promoted as an HIV prevention strategy. For those who are HIV infected, circumcision is only performed on the recommendation of an HIV clinician after an individual’s health status is assessed and the CD4 count > 350 mmol/L [27]. One participant had not been circumcised as he was informed at his clinic that HIV-infected males were not circumcised. Fearful of disclosure, this caused him some anxiety:

*‘There is a time when as a male, when you need to urinate, there is a saying amongst males that a man doesn’t urinate by himself. So, they ask why you aren’t circumcised, and you have to start creating a lie because you can’t just blurt out that it’s because you have HIV.’*

##### Participation in sport

**Fig. 5:**
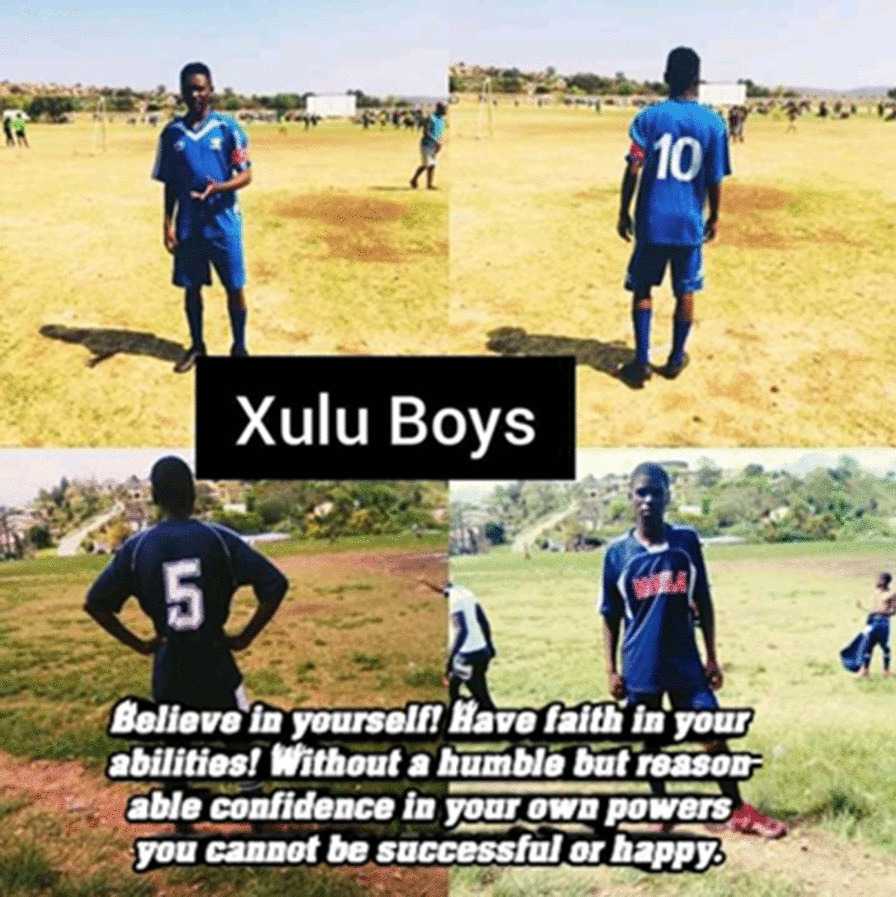
Football as an enabler of adherence

Sport was both a coping mechanism for a number of participants as well as facilitating their adherence to ART. The male participants all spoke about soccer and one female participant described her involvement in netball as an enabler. On participant shared a photograph (Fig. 5) of him and other members of his soccer team ‘The Xulu Boys’ saying:

*‘The reason why I took a picture of a football is because I am at my happiest if I am playing football. If I am playing football, I forget that I have this virus.’*

Sport not only helped the study participants forget their HIV status and feel ‘normal’ like their peers, but facilitated ART adherence, as to to play sport and play it well, ART adherence and consequent viral suppression is necessary.

#### Social domain 4: the clinic

The fourth social domain that had an impact on adolescent adherence was the clinic. Relationships and interactions with people in the clinic—including doctors, nurses, and social workers—were all reported as either helping or challenging adherence—often moderated through what participants perceived and described as “respect”. Physical and logistical aspects, such as the distance to the clinic and operating hours, were also reported as facilitators or barriers to adherence.

##### Health care providers

In the first FGD all participants who mentioned the clinic mentioned the care and support they received there. One participant spoke about the positive role social workers played, both in supporting her treatment journey, but also in supporting her as she struggled with her life’s issues. The quote below relates to their support for her treatment journey:

*‘…it’s not that I love them but the social workers here at the clinic and the counselling they provide here has been very helpful, I don’t want to lie. They are very kind; they are very nice, and they don’t shout at you. They ask what problem you have, and you tell them. They have thousands of ways in which they can assist you in and you just choose one that will work best for you. They are very friendly. You know when the doctor says I need counselling, I get very excited because I know that I will speak freely with them and they will try to assist me as best as they can without arguing with me.’*

A number of other respondents also spoke positively about social worker support and encouragement to take their ART more regularly:

*‘I spoke to the social worker not because of any bad occurrences, it was because the social worker wanted to commend me for taking my treatment properly and that my medical charts showed that I am adhering to treatment.’*

Another participant described how the doctor and nurse had shouted at him, but admitted they had reason to shout at him, as he had not been taking his treatment.

##### Knowledge and information about HIV and ART

In the second FGD a number of the participants felt that they had insufficient information about HIV and ART, and staff at the clinic were too busy to take the time to educate them. After responding to a number of HIV related questions in the FGD the researcher facilitating the FGD responded:

*Researcher: ‘You guys have made me start a class, maybe I should start teaching you all on Saturdays.’*

*Participant: ‘Maybe that’s going to help because the nurses at the clinic don’t have time.’*

*Participant: ‘Plus, I have lots of questions.’*

*Participant: ‘There should be a nurse who is going to come here every Saturday and teach us….and answer our questions.’*

*Participant: ‘That’s difficult because the clinics are always full, and you will feel like you are taking too much time if you start asking questions about this and that…’*

*Participant: ‘They really don’t have time at the clinic…’*

*Participant: ‘They are always busy’.*

One participant went on to say:

*‘It’s this thing of not getting adequate information about what the pills are for, so I would only take them when it suited me.’*

#### Adolescent experiences and sense of self

All the domains described above exerted an influence on adolescent engagement in HIV care. So, too, did aspects of the adolescents’ own experiences and identities, including perceptions of self, illness experiences (including HIV and non-HIV illnesses), knowledge base, side effects from ART, disclosure, substance use, hopes for the future, and socioeconomic stressors. Although these experiences were felt by the adolescent him/herself, they were often interpreted through the social domains described above. Each of these are described in more detail as well as the ways they contributed to or detracted from adherence to ART and engagement in care.

##### Knowledge and understanding of HIV and ART

**Fig. 6:**
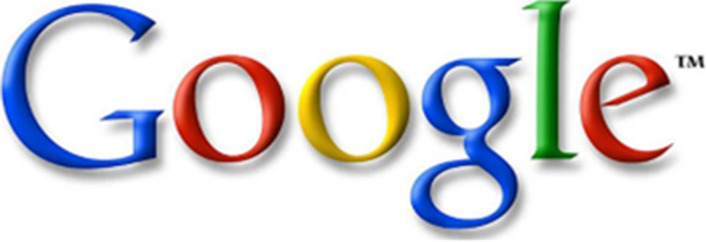
A photo of the google search engine—access to information is an enabler of adherence

Knowledge and understanding of HIV and ART facilitated adherence. Conversely, limited understanding and knowledge of HIV and ART undermined adherence. One participant took a screenshot of Google (Fig. 6) and said:

*‘If I could explain about Google, I can say that in our communities where we live, most people have wrong information about HIV. I don’t know if they twist the information or I don’t know, but the information that they have is different from the information that we have. Personally, I have used Google a lot to do my own research about this virus throughout my journey of living with it because some questions I felt like I couldn’t ask people so I would just look it up on Google.’*

However, knowledge about HIV and ART did not always enable adherence. Two participants who described knowing that ART was necessary to control the virus and prevent death were not taking their ART at the time of the study and appeared to be in a phase of denial:

*‘They do say that some people are able to live with this virus even if they don’t take the pills.’*

*‘I have this virus. I believe I am gifted because I have the power to control it. This virus doesn’t make a death sentence, all you must do is manage it so that you don’t die.’*

##### Experiencing the benefits of ART

Four participants described how their health improved after they restarted ART. Three participants had been very ill and were hospitalised, but their experience of the positive effects of ART led to improved levels of adherence. One of these four was hospitalised with his mother and although he recovered, she died of HIV in the hospital. The third participant experienced a significant improvement in her health after restarting ART, which also led to improved levels of adherence. The need to adhere to treatment was reinforced by the fear of becoming ill again and the fear of death. One participant described how during a period of poor adherence, he developed TB meningitis and spent 6 months in hospital. When asked if he would take his pills in the future he responded:

*‘Absolutely, because of what I saw in hospital. What I saw frightened me. People die in hospital.’*

A couple of participants described experimenting with not taking their ART to find out if it was necessary for them to keep taking their medication. One participant reported:

*‘Yes, I wanted to confirm if I am not taking these pills for the sake of taking them. Maybe I have been cured.’*

##### The side effects of ART

A number of participants took photos of their ART pills and described both ingesting and the side effects of ART as barriers to optimal retention in care. One participant described his diffculty in taking the pills saying:

*‘They are so big, if I take the pills I vomit.’*

Others described breaking the pills in two or crushing them and drinking them with warm water to facilitate ingestion, but these strategies did not really help. However, even more significant than the difficulty in ingesting the pills were the side effects they all experienced. One participant reported:

*‘I don’t take my pills, if I take the pills, they make me feel weak. They also affect my moods, I get agitated very easily. I try to avoid taking the pills……‘When I take these pills before I go to school in the mornings, I get to school tired, sometimes I suddenly just get sick. I get sick even at home, the pills make me tired and they make me want to sleep. If its hot, the pills make me nauseous.’*

*‘I sometimes do take my pills before I go to school and they make me feel sleepy. I get extremely fatigued when the teacher comes in the classroom during the first period.’*

Another participant described himself as a quiet person, but that ART affected his moods:

*‘Sometimes taking these pills make me aggressive and I feel like I could hit someone if I have taken them. They make me short-tempered. The pills affect my moods and I’m generally a very quiet person.’*

A further two participants experienced nauseau as a result of ART:

*‘Yes, the pills also make me nauseous but before I take them, I make sure that I eat first so that I don’t experience the nausea. If I take the pills without eating, it always seems as though I will throw up. But if I eat before taking them, nothing happens. I just go to school and feel fine.’*

*“I don’t want to lie I wasn’t taking my treatment. I wasn’t taking my pills because they made me feel exhausted, I would also get nauseous as a result I would lose my appetite.’*

A number of participants described how the practical assistance of supportive individuals in their lives helped them overcome both the difficulty in ingesting ART as well as its side effects.

##### The future

Thinking of the future generated ambivalence in our study participants. One the one hand was the fear of inadvertent disclosure, the difficulties involved in establishing intimate relationships and the risks associated with sub-optimal adherence, one participant expressed her fear of the future and its enabling effect on her adherence:

*‘So, I decided that I must take the pills consistently because when I start getting sick, my whole family loses hope. Even here in hospital, I don’t know if it was a scare tactic, but I was told that if I default the third regimen, they will issue me with a form that I would have to sign that states that I will no longer be given any other form of treatment until I die, that’s what they said. So, I decided that I will take them irrespective of the side effects.’*

However, on the other hand, participants spoke of their hopes and possibilities for the future. One participant hoped to become a nurse and another said:

*‘I want to reach my goals and fulfil my dreams. I want to be a music artist and there is one artist I would love to sing with.’*

**Fig. 7:**
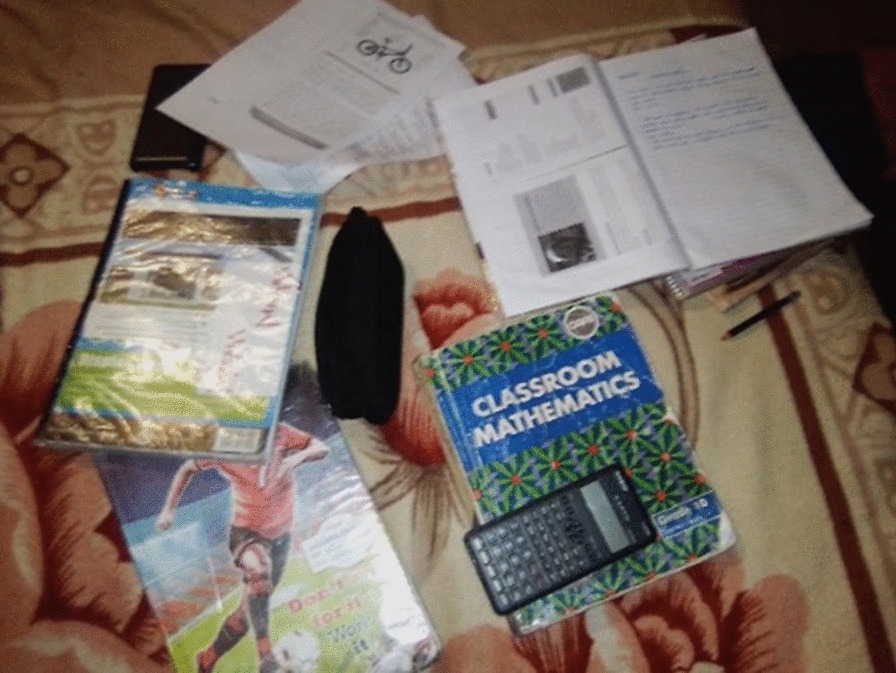
Studying a favorite subject at school as an enabler of adherence

A number of participants spoke of the importance and value of school and in having the right friends who wouldn’t lead them astray. One participant, who hoped to become a doctor, took photos of his school pens and pencils and his mathematics book, as these encouraged him to keep taking his treatment (Fig. 7):

*‘This is a picture of my school because I go to school every day because I know what I want out of life and without school, I know my options are very limited. The next picture is a picture of my books and its only math books because that is my favorite subject.’*

## Discussion

Sustaining optimal ART adherence for ALHIV is a major challenge. In addition to the challenges to ART adherence facing all ALHIV, adolescents who acquired HIV perinatally, often have the additional challenge of absent parents and an unstable and unsupportive home environment. In our study several participants described how unstable home environments and travelling and staying with different sides of the family had contributed to sub-optimal adherence during their life course. These findings are supported in the literature on ALHIV where being orphaned or having an unstable home environment is associated with poor adherence due to a lack of effective guidance, supervision and inconsistent care [5, 28, 29]. An unstable or unsupportive home environment also contributes to psychological distress, increased sexual vulnerability, and high rates of risk taking, which further undermine adherence [5, 28, 29].

We found that the fear of disclosure was another significant contributor to sub-optimal adherence. The fear of discrimination and stigmatisation from inadvertent disclosure impacted on school attendance, relationships as well as anxiety and reluctance to engage in intimate relationships. With regards to school attendance, as the ART clinic was only open during school hours, participants who had not disclosed their HIV status at school did not attend school on that day. Most participants described disguising their ART by repackaging it and ensuring no-one saw them taking their medication at school. Similar findings have been reported in other studies where issues with schooling include absenteeism due to illness or clinic appointments, a lack of disclosure of HIV status to school officials, and stigmatisation from classmates and teachers [5, 30]. With regards to relationships and intimate relationships, several participants expressed their fear and inability to establish intimate relationships, as this would involve informing their partner of their HIV status. Disclosure in intimate relationships is often reported as a factor contributing to poor adherence [31]. Although described under the school domain, fear of disclosure was one of many issues which intersected with more than one domain.

Many participants in our study reported the inflexibility of the ART regimen as an obstacle to adherence. The unique behavioural and lifestyle characteristics of adolescents, their desire for conformity, high rates of risk taking and the fear of being treated differently and being stigmatized because of their HIV status are incompatible with the nature of ART medication and inflexibility required for optimal adherence [32–34]. Most ALHIV adolescents want to ‘feel normal’, like their HIV-uninfected peers, but ART is a daily reminder that they are HIV-positive and different from their peers [14, 32].

In spite of these barriers, there were multiple reports of factors in all four social domains and in the adolescent’s own experiences that contributed to their ability to constructively deal with their HIV status and ART—something that is often referred to as “resilience” [35]. Other studies have identified resilience as important in the narratives of adolescents living with HIV [36], and when objective measures of resilience are low this is associated with poor outcomes in adolescent populations [37]. We have identified several interventions in each of the social domains and in adolescents’ perceptions, experiences and sense of self which could build the resilience of ALHIV (Table 2).

**Table 2:** Potential programmatic interventions to support adolescents living with HIV

Domain	Facilitators and barriers reported by participants	Potential programmatic intervention
Domain 1: The Household		
	Facilitators: Supportive family members	Help adolescents identify one supportive individual in the household
	Barriers: Absent parents, unstable housing, lack of discussion on HIV, socioeconomic stressors	Cultivate adult mentorship if parents absent, carry small supply of ART if household unstable to improve access to once daily ART, role playing on facilitating discussions on HIV and ART with the adolescent leading, provide socioeconomic support or incentives for the adolescent, cultivate family and household models of adherence support
Domain 2: The School		
	Facilitators: School content, supportive friends, supportive teachers	Identify positive school experiences as motivators for future and adherence, help adolescents identify supportive peers and adults, peer training programs
	Barriers: Schools discussions on HIV, fear of inadvertent disclosure, missing school, stigma	Role play with adolescents on how to handle discussions of HIV in the classroom, train adolescents on how they can respond to potential disclosure situations, minimize school absences to attend clinics (see clinic section below), build self-esteem among adolescents living with HIV
Domain 3: The Larger Community		
	Facilitators: Supportive relationships, sports	Help adolescents identify potential supportive adults and peers, identify positive community institutions and events (i.e. sports, church) and incorporate these into adherence counseling
	Barriers: Fear of inadvertent disclosure, missing “rites of passage”, stigma	Role play with adolescents on how to handle possible disclosure, identify strategies for participation in important rites of passage, build self-esteem among adolescents living with HIV
Domain 4: The Clinic		
	Facilitators: Supportive providers	Train providers in needs of adolescents and develop adolescent honest and friendly models of care (i.e. peer support, mentorship with adults who are on ART, etc.)
	Facilitator: Clinic-based peer support group	Work with motivated charismatic adolescents to set up clinic-based peer support groups
	Barriers: Clinic hours, lack of information about HIV and ART	Have flexible or evening clinic hours or dedicate one afternoon a week after school for adolescents, provide refresher training on HIV and ART using media/messaging that is adolescent friendly
Adolescents’ perceptions, experiences and sense of self		
	Facilitators: Internalized knowledge about HIV and ART, experiencing benefits from ART	Actively listen to adolescents express their understandings of HIV to develop individual training on HIV/ART, remind adolescents about positive benefits of being on ART, pair adolescents with adults on ART who have done well, peer support
	Barriers: Side effects from ART	Screen for possible side effects and develop empowering management strategies for common problems

ART: antiretroviral therapy

Ongoing education is important and needs to be dynamic to address the changing needs and challenges facing ALHIV. One study reported perinatally infected ALHIV appreciate honest communication with health care providers as they negotiate new challenges, depression and complicated grief [38]. Many of our participants did not understand how they had contracted HIV or the pathways to vertical transmission, needing more reliable and comprehensive information on vertical transmission, prevention of mother to child transmission and reproductive health options. This information could inform a discussion on disclosure of HIV status, how to disclose in an intimate relationship and how to reduce the risk of transmission during physical intimacy.

In addition to more reliable information, counselling and support models that focus on fortifying adolescents’ coping skills and their perceptions of themselves as strong and empowered individuals could help engage them in ongoing HIV care and adherence with ART [39]. Several participants in our study were isolated from their peers. A South African study reported that adolescents valued clinic-based support groups [40], and there is growing evidence that if run well, clinic-based peer-support groups improve adherence [41, 42]. Groupwork is a powerful method of reaching young people who enjoy the company of their peers, and shared experiences can facilitate understanding [40]. Moreover, in resource constrained situations, they are a cost-effective strategy. As our study and others have reported, non-verbal therapies such as drawing, writing and photovoice appeal to adolescents and could be used as counselling strategies [43]. These counselling and support models are most beneficial if offered as part of differentiated service delivery models with peer network development, something that our results also support.

This study has important findings that merit further study and validation. There are, however, several limitations. First, all the participants in this study acquired HIV through the perinatal transmission route. This is not surprising given the age range of our study participants, as it was not until the last decade that ART became widely available in South Africa. It is, however, a limitation of this study, in that adolescents who acquire HIV via sexual transmission may have different experiences than those who acquired it via peri-natal transmission. It will be important to include such adolescents in future explorations of their treatment journey with ART. Second, only 25% of the adolescents who could have potentially participated in this study were able to be contacted. This population likely represents one of the most stable groups of adolescents in HIV care at the clinic, and thus their experiences may not reflect those of other adolescents with potentially even greater barriers to remaining in care. Third, the use of PVM was meant to facilitate open discussion with ALHIV but there may have been some gender and age issues with the adolescents and the study investigators that led to biased reporting from participants. Fourth, FGDs may have led to peer pressure and different behaviours being reported by participants than if individual interviews had been done. While this is true of any qualitative study, the unique desire for peer approval among adolescents may have made this methodology more prone to validity bias than with other populations. Finally, our study did not assess if saturation was reached but rather we interviewed the entire population that was willing and able to participate. As a result this was a relatively small sample size with a predominantly male population and further investigation is warranted as some barriers and facilitators of adherence may not have been reported. Given the paucity of data on the experiences of this population, however, we felt our results were important to share despite the small sample size.

## Conclusion

ALHIV face multiple challenges with their lifelong therapy that are not adequately addressed through current models of adherence support. Providing them with skills in which they can navigate the different social domains in which they operate could contribute to strengthening existing models of adherence support. Helping adolescents define and see themselves as resilient may be one useful way they can remain engaged in HIV care and adherent to ART that merits further exploration in this vulnerable population.

## Supplementary Information

**Additional file 1. **Detailed study methodology.

## Data Availability

The datasets used and/or analyzed during the current study are available from the corresponding author on reasonable request.
