# Supplementary material for: ‘If I am playing football, I forget that I have this virus’: the challenges and coping strategies of adolescents with perinatally acquired HIV in KwaZulu-Natal, South Africa
Source: BMC Infect Dis. 2022 Oct 21;22:796. doi: 10.1186/s12879-022-07780-x (PMC9587556; doi:10.1186/s12879-022-07780-x)
Supplement: Supplementary file 1 — Additional file 1. Detailed study methodology. [file 12879_2022_7780_MOESM1_ESM.docx]

**Annexure 1:**

**Section A: Participant-generated Visual Methodology (PVM)**

The 14 adolescent participants enrolled in the study were divided into two groups, with seven in each group. The Participant-generated Visual Methodology (PVM) research process was run with each group. This process included a training workshop, a week during which images were captured and a final Focus Group Discussion (FGD). Details of each stage in the research process are described below.

**Training workshops**

The PVM training workshops were on a Saturday morning so that those who were at school, or had other commitments during the week, could attend. Each training workshop lasted just less than an hour and a half. The workshops were held at a clinical research site within the grounds of the hospital where the ART clinic is based.

At each workshop, after the study team and participants introduced themselves, the study team: 1) described the background and rationale of the study; 2) discussed the need for complete confidentiality about the training, study processes and FGDs as some participants may not have disclosed their HIV status to their family or friends; 3) introduced the idea of capturing experiences in an appropriate manner using PVM; 4) facilitated a discussion to stimulate ideas of what photographic images or drawings or paintings would be suitable for the overall aim of the study; 5) provided training on taking photographic images in the communities in which the participants lived; and 6) provided participants with cell phones with cameras and drawing and painting materials.

In addition, participants were given a one-page ‘Participant Guidelines for Research Activity’ document explaining how the photographs should be taken (This guideline on pages 3 and 4 of this annexure.) In the document study participants are asked to capture images representing their experiences of living with HIV, together with the challenges encountered and support they received during their treatment journey that impacted their retention in care.

To minimise risks to participants, we did not make use of expensive cameras, but instead provided participants with inexpensive cell phones with which to take photographic images. Drawing and painting materials were also provided by the study team. During training we emphasized the importance of not taking photographic images which could put participants at risk, i.e., photographs in which an individual could be recognised were not to be taken if the individual was unknown to them. We also discussed with participants that the photographs and drawings/paintings would be the data generated by this study component and that they had a right not to share photographs/drawings/paintings that they do not want included in the analysis.

**The week after the training workshop - taking photographic images, drawing or painting**

Following the workshop, participants were given a week to take photographic images, draw or paint pictures. This allowed participants time to think about the images they had captured and allow for deeper reflection of their own experiences. In addition, we hoped this would facilitate sharing by those who are typically quieter during traditional FGDs or interviews.

Each day during the week after the training course participants were phoned by a member of the study team to ensure they had not run into problems and encourage them to keep taking and collecting images or with their drawing or painting.

The day before the FGD a member of the study team picked up the cell phones and drawings. Each participant was asked to select three images they were comfortable to share with the other participants at the FGD. These were printed and enlarged and returned to the participants at the FGDs. The images selected were downloaded onto a password protected computer.

**Focus Group Discussion around photographic images/drawings/paintings**

To complete the research process FGDs were held. Each FGD took between two and a half and three hours and included a break for a refreshments in the middle. The printed images were attached to the walls of the venue and in turn, each participant showed their images/drawings to the group and described why they had chosen these particular images. This process of sharing led to a discussion which was facilitated by a member of our study team with a decade of qualitative research experience. The FGD was audio-recorded and as it was conducted in the local language (isiZulu), was then translated and transcribed.

**Section B: Focus Group Discussion Guideline**

Welcome and introductions

Reminder of the aim and rationale of the study

Sharing of a photo/drawing or painting which best describes each participant followed by discussion

Sharing of a photo/drawing or painting which best describes each participants experience of living with HIV, followed by discussion

Refreshment break

Sharing and discussion of what has helped and supported each participant to stay on treatment and visit the clinic regularly.

Sharing and discussion of what has made it more difficult for each participant to visit the clinic and take treatment regularly.

Wrapping up and Closure


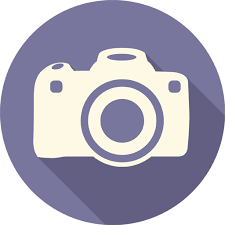
Section C: Participant guidelines for research activity


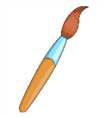


Thank you so much for agreeing to participate in this study. We hope to learn from you, through your photographs/drawings/paintings about your experience of being a young person living with HIV in this community, what and who has helped you visit the clinic regularly and stay on treatment and what challenges you have had to overcome to stay on treatment. Below we have provided some guidelines that we would like you to keep with you as you are taking your photographs/drawing or painting. We would like you to take 4 photos in response to each of these topics below. Please use the cell phone we have provided you with to take photos. Or, if you prefer you can draw or paint to illustrate your experiences with the materials we have provided here. If you chose to draw or paint you may need less than 4 drawings/paintings for each topic


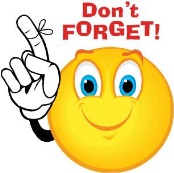


# Some important things to remember

1. Make sure you understand how your camera works.
2. Make sure you think about these topics before you start taking your photos.
3. Take a day to walk around your community to identify the things you want to take photos of, draw or paint in relation to the topics below.
4. Try not to take photos of people’s faces or things that would show identifiable information of the person or places (for example, the name of a person’s tavern or shop).
5. You can ask someone to take a photo of you, but it must be someone you know and trust.
6. Try to take photos during the day. If you want to take a photo at night or when the light is not so bright, you will need to check the photo is still clear. You may need to use the flash.
7. Take photos of things while you are ‘standing still’, otherwise they maybe blurred or not so clear.
8. It is OK to pre-arrange/stage an image- for example if you gather some things and put them in one pile and if you worry that a picture will be too risky (e.g., drinking alcohol)
9. Please try not to take more than one picture of the same thing
10. ENJOY the activity!

# Topics to respond to:

Please make sure that you respond to ALL the topics below. Tick off the topics you have completed in the space provided below.


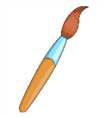

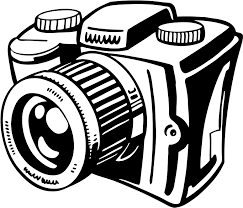


| Topics to cover | Tick when done |
| --- | --- |
| 1. Please take 4 photos/draw/paint something which best describes who you are. |  |
| 1. Please take 4 photos/draw/paint what best describes your experience of living with HIV in your community. |  |
| 1. Please take 4 photos/draw/paint what has helped and supported you as a PLHIV to stay on treatment and visit the clinic regularly. |  |
| 1. Please take 4 photos/draw/paint what has made it more difficult for you to visit the clinic and take treatment regularly. |  |

**
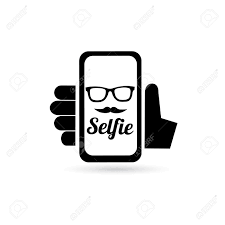

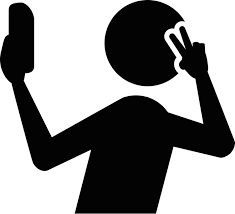

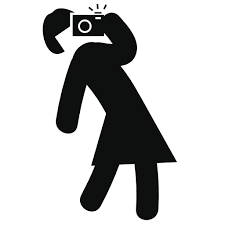
Thank you for participating!**
